# Palatal rugae assessment using plaster model and dental scan: a cross-sectional comparative analysis

**DOI:** 10.3389/froh.2024.1456377

**Published:** 2024-12-20

**Authors:** Laura Roselli, Federica Mele, Carmela Suriano, Valeria Santoro, Roberto Catanesi, Massimo Petruzzi

**Affiliations:** ^1^ Dental School, University of Bari, Bari, Italy; ^2^ Legal Medicine Section, University of Bari, Bari, Italy; ^3^ Private Practice, Andria, Barletta-Andria-Trani, Italy

**Keywords:** palatal rugae, oral scan, oral impression, personal identification, forensic odontology

## Abstract

**Objective:**

Due to their consistent and individualistic patterns, palatal rugae (PR) are used in forensic dentistry as an ancillary method for personal identification. This study aimed to compare the impression of the PR obtained with the classic alginate impression and casting of the plaster model with the impression of the palate made with an intraoral scanner. Both impressions were compared with each other and with the photograph of the palatal rugae.

**Materials and methods:**

In this study, 19 patients (6 men, 13 women; mean age 28.6 years) were selected. Two different impressions were taken from the maxillae of the participants: a conventional impression using alginate impression material, and an optical impression using an intraoral scanner. The impressions obtained were compared with each other and with the photograph of the palatine rugae of each enrolled patient, using FaceComp™ software. The parameters assessed included absolute and relative distances, perimeters, areas, shape factors, and moments. The statistical analysis was conducted using Python 3.9.

**Results:**

The data from digital and plaster models were comparable across all six parameters used by the software. The coefficients of correlation and determination were strong to very strong for all six parameters assessed, with no statistically significant differences detected between the two methods of palatal rugae impression.

**Conclusion:**

Both digital and traditional methods were equally reliable in capturing palatal rugae patterns. The use of FaceComp™ software facilitated accurate comparison and personal identification through the alignment of the preidentified landmarks. Further studies are required to enhance the speed and precision of image acquisition and comparison for broader application in personal identification.

## Introduction

Palatal rugae (PR) are asymmetrical and irregular mucosal elevations located in the anterior third of the palate. They consist of the lateral membrane of the incisor papilla arranged transversely from the palatine raphe in the mid-sagittal plane (1). These rugae emerge around the third month of intrauterine life, originating from connective tissue covering the palatine process of the maxillary bone (2). Their development and growth involve reciprocal control through interactions between epithelial and mesenchymal cells, expressing specific molecules in the extracellular matrix during development (3). While their size may change due to palatal growth, their shape remains constant (4). Functionally, PR contribute to oral deglutition and enhance the relationship between food and taste receptors on the tongue's dorsal surface (5). Individual rugae patterns exhibit unique configurations and remain unchanged with growth, analogous to a fingerprint (6).

PR exhibit resilience against deterioration, often reappearing after trauma or surgical interventions, and are protected by surrounding oral structures. In addition, they remain unaffected by external factors such as chemicals, heat, disease, or trauma (7, 8).

Rugoscopy refers to the study and analysis of PR, involving an examination of their patterns, shapes, and characteristics (9). This specialized field is primarily used in forensic dentistry and personal identification, utilizing the unique nature of PR to establish an individual's identity (10).

In cadaveric identification, analyzing PR is particularly beneficial in edentulous patients where other dental identification methods are impractical. Hence, PR serve as reliable identifiers in human identification scenarios involving carbonized, decomposed, or disfigured corpses, contingent upon *antemortem* records (11).

They also serve as stable reference points for overlaying three-dimensional virtual models before and after orthodontic treatment. Some researchers have explored their use as reference points for measuring tooth movement, even as a substitute for cephalometric superimposition (12).

In clinical practice, the study of PR involves taking an impression, usually in alginate, followed by casting a plaster model that highlights the rugae. In recent years, with the advent of digital dentistry, it has become possible to scan dental arches using an intraoral scanner (IOS) and obtain a three-dimensional (3D) reconstruction, including the PR (13).

The use of an IOS for PR analysis represents a significant advancement in dental imaging technology. Unlike traditional methods that rely on physical impressions and subsequent manual measurements, an IOS offers a non-invasive, highly accurate, and efficient means of capturing detailed images of the palate (14). This modern approach not only enhances the precision of PR analysis but also improves dental office procedures and streamlines the comparative process. The novelty of this application lies in its ability to provide real-time digital impressions, facilitating more detailed and reproducible assessments of PR compared to conventional techniques (15). Previous studies have largely relied on analog methods, which are often prone to inaccuracies due to manual handling and potential distortions during the impression-taking process. These traditional approaches also involve significant time and workload, potentially impacting the overall efficiency of dental practice (16). In contrast, the current research addresses these limitations by employing IOS technology to overcome the constraints of previous methods.

The aim of this study was to evaluate whether the digital impression of the PR was reliable and comparable to that obtained with alginate, or whether there were differences between the two impression methods.

## Materials and methods

### Ethics

This study was conducted in accordance with the Ethical Principles for Research Involving Human Subjects, as outlined in the World Medical Association (WMA) Declaration of Helsinki, Seventh Revision (64th WMA General Assembly, Fortaleza, Brazil, October 2013). In addition, it received approval from the Local Ethics Committee IRCCS Oncology Institute “Gabriella Serio” operating at the University of Bari “Aldo Moro” (study protocol approval no. 1316/CE).

### Recruitment of participants

In June 2023, a cohort of 19 consecutive adult volunteers was included in this study. Individuals were included if they had at least four upper incisors, no lesions on the palate, and did not wear orthodontic or prosthetic appliances. Patients with previous cleft palate were excluded.

### Sample size calculation

The sample size was calculated to compare the accuracy of palatal rugae recordings using an IOS versus alginate impressions. A significance level of 0.05 and a power of 90% were selected. The minimum detectable difference between the two methods was set at 0.05 mm, with an estimated standard deviation of 0.1 mm. Using the formula for a paired *t*-test: $n=\frac{{\left(Z_{\alpha /2}+Z_{\beta }\right)}^{2}\cdot \sigma^{2}}{d^{2}},\;\mathrm{where}\;Z_{\alpha /2}=1.96\;\mathrm{and}\;Z_{\beta }=1.28$, the calculated sample size was 18 patients.

### Palatal rugae imaging

Each participant's palate was photographed using a Digital Camera D7200 (Nikon Corp., Tokyo, Japan), Macro Ring Lite (Sigma EM-140 DG; Nikon Corp., Tokyo, Japan), and an intraoral photo mirror (Hager & Werken GmbH & Co., Duisburg, Germany). For each patient, a traditional alginate impression (Hydrogum 5; Zhermack SpA, Badia Polesine, Italy) of the upper arch was taken, from which, a plaster (Schein Dental, Eschborn, Germany) model was subsequently created. At the same time, the palatal area of each participant was scanned using an iTero Element Flex IOS (Align Technology B.V., Amsterdam, the Netherlands) in a zig-zag scanning pattern, starting from the incisive papilla and finishing at the border of the hard and soft palate. The same dentist (CS) who was experienced with this specific system made all clinical photographs, alginate impressions, and scans.

To compare the plaster model and the scanned model, two-dimensional images of each were captured as follows: the plaster model was photographed to obtain a high-resolution two-dimensional image in TIFF format. At the same time, the digital model of the palate, originally in STL format, was converted to TIFF format for the comparative analysis.

### Landmarks

The images of each patient (clinical palate, plaster model, and scanned palate) were superimposed and rotated using Adobe Photoshop software (Version 22.x; Adobe Systems, San Jose, CA, USA): the grid function was employed to achieve the highest possible accuracy. Five parallel lines were drawn for each patient image: the first traversed through the interincisal line, while the remaining four lines passed through the central point of the collar of each upper incisor (central incisor and lateral incisor), aiming to establish standardized landmarks for subsequent identification.

**Figure 1:**
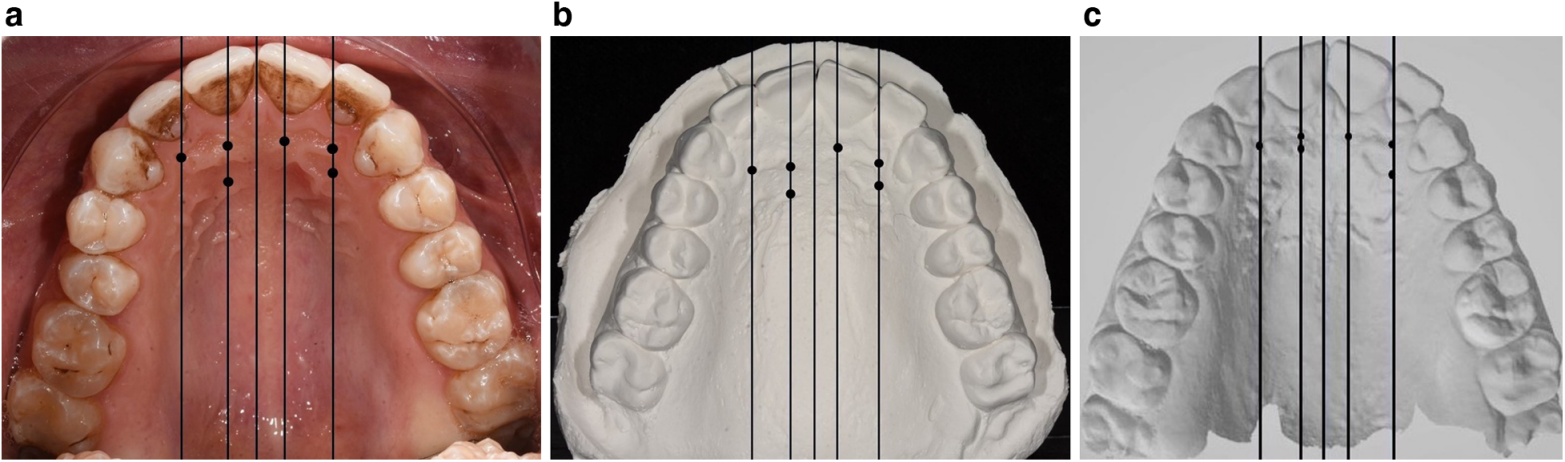
The six identified landmarks on the clinical photograph **(a)**, plaster model photograph **(b)**, and scanned model **(c)**.

After that, six different points were selected for each palatal image in the subsequent sequence: (1) intersection point of the line passing through the left upper central incisor (2.1) with the first PR; (2) intersection point of the line passing through the left upper lateral incisor (2.2) with the first PR; (3) intersection point of the line passing through the right upper central incisor (1.1) with the first PR; (4) intersection point of the line passing through the right upper lateral incisor (1.2) with the first PR; (5) intersection point of the line passing through the right upper central incisor (1.1) with the second PR; and (6) intersection point of the line passing through the left upper lateral incisor (2.2) with the second PR. The identified landmarks for each palatal image are summarized in Figure 1.

The same expert forensic doctor (FM), a regular user of the FaceComp™ software, performed the landmark tracing.

### FaceComp™ software for images comparation

FaceComp™ (NewFaceComp.exe) is a software developed by the Polytechnic University of Bari (Italy) that allows the matching of two geometrical figures through preselected points identified in the photograph.

Each marked image was uploaded to FaceComp™ software, which automatically supplies measurements on absolute distances, relative distances, shape factors (a value that numerically describe the shape of a particle, independent of its size), moments (a quantitative measure of the shape of a function), perimeter, and area of a polygon obtained by joining the landmarks.

The parameters of the algorithms were calculated as follows:

Letting *x_i_* and *y_i_* be the generic coordinates of a point, and *I*, J, and *K* the points of a generic triangle, and *p_ijk_* the perimeter of the triangle, the area can be obtained in the following way:

$$\mathrm{area}\_\mathrm{tri}\;=\;1/2\mathrm{Abs}\left(\begin{array}{c} x_{i\;\;\;}y_{i}1\\ x_{j}y_{j}1\\ x_{k}y_{k}1 \end{array}\right)$$

where Abs is the method for the solution of a general linear algebraic system.

The related compactness index is as follows:

$$\mathrm{comp}\_\mathrm{ind}\;=\;\mathrm{area}\_\mathrm{tri}/p2_{ijk}$$

This index, as a shape factor, is a non-dimensional value that describes the irregularity of the geometric figure represented.

The FaceComp™ software includes different functions, such as interactive positioning of landmarks for morphometric analysis, digitization, visualization of parameters for each image, and automatic calculation and presentation of comparative data. The software calculated the coefficient of correlation and the coefficient of determination for each comparison made between palatal photograph, plaster model, and the oral scan.

### Statistical analysis

The statistical analysis was conducted by arranging the data in an Excel database. The analysis was performed using Python 3.9 through the SciPy statistical library.

The correlation coefficient (*r*) and the determination coefficient (*R*^2^) were used for the evaluation of each of the six parameters used by the FaceComp™ software.

For *r* values <0.3, the correlation was considered weak; for 0.3 < *p*-values < 0.7, the correlation was considered moderate; and for *p*-values >0.7 the correlation was considered strong.

For *R*^2^ values equal to 0, no relationship was considered: for 0 < *R*^2^ < 0.2 the relationship was considered weak; for 0.2 < *R*^2^ < 0.4 the relationship was considered moderate; for 0.4 < *R*^2^ < 0.6 the relationship was considered strong; for 0.6 < *R*^2^ < 0.8 the relationship was considered very strong; and for 0.8 < *R*^2^ < 1.0 the relationship was considered perfect.

The *r* and *R*^2^ values obtained from each comparison were statistically compared using the Wilcoxon–Mann–Whitney test. Statistical significance was established for values with *p* < 0.05.

### STROBE checklist

The present study adheres to the Strengthening the Reporting of Observational Studies in Epidemiology (STROBE) checklist. The checklist is included as Supplementary Material.

## Results

There were 19 enrolled patients aged 18–51 years (mean age 28.6 years) with a M:F ratio of 6:13.

Table 1 shows the data related to the FaceComp™ comparison between the PR images obtained from clinical photographs, plaster models, and the IOS.

**Table 1:** Palatine rugae comparison between digital impression, analogical impression, and clinical photograph.

FaceComp™ Parameter	Coefficients mean ± DS (range) median (IQR)	Comparison of palatal photograph and digital scan	Comparison of palatal photograph and plaster model	Comparison of digital scan and plaster model
Absolute distances	Coefficient of correlation	0.9845 ± 0.0153 (0.9692–0.9998) 0.9894 (0.9791–0.9961)	0.9826 ± 0.0344 (0.9482–1.017) 0.995 (0.9889–0.996)	0.984 ± 0.015 (0.969–0.999) 0.984 (0.9535–1.0145)
	Coefficient of determination	0.9696 ± 0.0298 (0.9398–0.9994) 0.979 (0.9586–0.9922)	0.9666 ± 0.064 (0.9026–1.0306) 0.9901 (0.9779–0.9919)	0.968 ± 0.030 (0.938–0.998) 0.968 (0.9085–1.0275)
Relative distances	Coefficient of correlation	0.9198 ± 0.091 (0.8288–1.0108) 0.946 (0.897–0.9893)	0.9293 ± 0.0836 (0.8457–1.0129) 0.9821 (0.8698–0.988)	0.898 ± 0.080 (0.818–0.978) 0.927 (0.8045–1.0495)
	Coefficient of determination	0.8539 ± 0.1561 (0.6978–1.01) 0.8949 (0.8049–0.9787)	0.8703 ± 0.1488 (0.7215–1.0191) 0.9646 (0.7566–0.9761)	0.813 ± 0.140 (0.673–0.953) 0.859 (0.646–1.072)
Perimeters	Coefficient of correlation	0.9769 ± 0.0245 (0.9524–1.0014) 0.9852 (0.9711–0.9945)	0.9765 ± 0.0403 (0.9362–1.0168) 0.9928 (0.9805–0.9959)	0.978 ± 0.019 (0.959–0.997) 0.980 (0.944–1.016)
	Coefficient of determination	0.955 ± 0.047 (0.908–1.002) 0.9705 (0.943–0.9891)	0.9551 ± 0.0748 (0.8803–1.0299) 0.9857 (0.9613–0.9918)	0.956 ± 0.036 (0.92–0.992) 0.960 (0.891–1.029)
Areas	Coefficient of correlation	0.6639 ± 0.3042 (0.3597–0.9681) 0.7045 (0.4802–0.9041)	0.6907 ± 0.3135 (0.3772–1.0042) 0.7902 (0.6562–0.9063)	0.615 ± 0.303 (0.312–0.918) 0.584 (0.0965–1.0715)
	Coefficient of determination	0.5284 ± 0.3551 (0.1733–0.8835) 0.4963 (0.2316–0.8174)	0.5702 ± 0.3109 (0.2593–0.8811) 0.6244 (0.4307–0.8216)	0.466 ± 0.332 (0.134–0.798) 0.342 (0.134–0.818)
Shape factors	Coefficient of correlation	0.7488 ± 0.2819 (0.4669–1.0307) 0.8745 (0.6593–0.9435)	0.721 ± 0.2847 (0.4363–1.0057) 0.8119 (0.5484–0.9318)	0.717 ± 0.188 (0.529–0.905) 0.722 (0.4385–1.0055)
	Coefficient of determination	0.6361 ± 0.3094 (0.3267–0.9455) 0.7648 (0.4347–0.8907)	0.5966 ± 0.3006 (0.296–0.8972) 0.6591 (0.3011–0.8684)	0.547 ± 0.266 (0.281–0.813) 0.521 (0.124–0.918)
Moments	Coefficient of correlation	0.9994 ± 0.0015 (0.9979–1.0009) 0.9998 (0.9997–0.9999)	0.9997 ± 0.0004 (0.9993–1.0001) 0.9998 (0.9997–0.9999)	1.000 ± 4.091 × 10^−4^ (9,995.909 × 10^−4^ –10,004.091 × 10^−4^) 1.000 (0.9995–1.0005)
	Coefficient of determination	0.9988 ± 0.003 (0.9958–1.0018) 0.9996 (0.9994–0.9998)	0.9994 ± 0.0008 (0.9986–1.0002) 0.9997 (0.9993–0.9999)	0.999 ± 8.172 × 10^−4^ (9,981.828 × 10^−4^ –9,998.172 × 10^−4^) 1.000 (0.9985–1.0015)

Analyzing the six parameters used by the FaceComp™ software to compare the images obtained yielded strong to very strong values of relationship.

The *r* and *R*^2^ values obtained from the comparison between the clinical photograph and the plaster model and between the clinical photograph and the digital impression did not show any statistically significant differences (*p* > 0.05).

## Discussion

The PR impressions obtained using classic alginate or an IOS showed no differences when compared with each other and with the clinical photograph of the PR.

The PR impression obtained through the classical method (using alginate and subsequent plaster model) is as reliable as that obtained with an IOS. Both methods showed no statistically significant difference when compared to the photograph of the patient's PR.

These data hold several implications, both in clinical practice and forensic medicine. Clinically, the efficiency and speed of digital impressions far surpass those using alginate, which necessitates longer execution times (a palatal scan can be completed in 18–22 s), even though both methods are equally reliable (17). In the field of forensic medicine, the digital impression of PR remains an additional method for personal identification. Several studies have highlighted how rugoscopy can determine an individual's sex or racial characteristics (18, 19). Moreover, the acquisition and digitization of PR enables a swifter and more effective exchange of data within the scope of teledentistry.

Mohammed et al. had previously demonstrated the possibility of utilizing dedicated software to overlay images of PR with those of plaster models, ensuring identification with 100% accuracy (20). Simon et al. demonstrated the ability to differentiate between two monozygotic twins by comparing their acquired PR through an intraoral scan. This study was the first to employ an IOS for rugoscopic analysis in the field of forensic medicine (17).

More recently, Bjelopavlovic et al. have demonstrated that PR scanned with an IOS can be considered an important method for identification in forensic medicine (21).

As mentioned, the digital impression represents a significant advantage in efficiency over the conventional impression.

To the best of our knowledge, no prior study has compared the congruence and reliability between impressions taken with alginate and plaster cast versus digital impressions of PR. In addition, the present study enables a quantitative rugoscopic analysis rather than the solely qualitative evaluations that are typical when evaluating PR (7).

The FaceComp™ software used in this study has proven effective for personal identification by comparing an individual's smile photographs with their plaster impressions (22).

However, the current study has limitations related to the small sample size and study design, which may limit the generalizability of its findings. Another limitation of this study is the lack of accuracy assessment for palatal scans using different IOSs. Variability in the digital oral scanning process can result from several factors, including operator technique, patient movement, and the specific scanning system used (23, 24).

Inconsistent lighting conditions or intraoral obstructions may also introduce errors, affecting the accuracy and reproducibility of digital impressions (25).

These sources of variability highlight the need for standardized protocols and training to ensure consistent, high-quality results in clinical practice.

PR patterns are considered unique to each individual, making them highly valuable in forensic identification. The use of IOSs for PR impressions, combined with image analysis and comparison software, has proven to be effective and reliable for comparative evaluation (26). Further studies are necessary to refine and speed up the image acquisition process, potentially through the use of dedicated scanners capable of processing and comparing PR images and comparing PR images against a database or existing records.

According to Jedliński et al. (27), “IOS are a modern, adequate, and increasingly accessible means for capturing and imaging the appearance of oral tissues*.*” Furthermore, they anticipated new applications of digital impressions in dental practice, as described in the present study.

As technology continues to evolve, the integration of IOSs into routine dental practice and forensic investigations is expected to increase, further establishing their role as a valuable tool in PR comparison and analysis.

## Data Availability

The raw data supporting the conclusions of this article will be made available by the authors, without undue reservation.
